# Dr. Kinglake, on the Cooling Treatment of Burns, &c

**Published:** 1806-11

**Authors:** 


### *To the Editors of the Medical and Physical Journal.*

Gentlemen,

Will you do me the favour of inserting in the next Number of the Medical and Physical Journal, the following Reply to an assertion of Mr. Ring's, published in No. xcii. p. 325, of that useful publication.

Yours, respectfully,

As in all controverted points the ultimate appeal legitimately lies with facts, you will see the propriety of publishing, at your earliest convenience, the enclosed cases, confessedly confessedly transmitted to me in support of the opinion I have lately endeavoured to establish respecting the superior advantages of the cooling treatment of burns and scalds. Dr. Kentish has felt no difficulty in ascribing common instances of seeming benefit from the cooling remedy, to the "salutary influence of nature triumphing over the interference of art." Whether the cases here submitted to the public are of that description or not, the unbiassed reader will be competent to determine.

To add to the practical value of these facts, I have judged it expedient to quote a few instances of similar efficacy from topical cold, recorded in Dr. Stock's Medical Collections of the Effects of Cold; a work, abounding with important information on the benefit resulting from the external application of cold water in various diseases.

*A Letter and Cases, communicated to* Dr. Kinglake *by* Dr. Evans, *on the Advantages of the cooling Treatment of Burns and Scalds.*

" Ketley, near Wellington, Shropshire, Aug. 28, 1806.

"Sir,

" From the very able manner in which you have defended the treatment of burns by the application of cold water, I am induced to take the liberty of sending you a few facts (selected out of a great number) to corroborate the practice you recommend. Perhaps, few medical men are in so extensive a line of curative surgery as myself, having for many years had the care of eleven or twelve hundred men and boys employed in the large iron works, and coal mines in this neighbourhood. Dr. Kentish's terebinthinate mode of treatment I disapprove of; it is particularly objectionable in burns of the face, on account of the eyes; and many person's skins would be considerably irritated by it, which I have had evident proofs of. There are many advantages attending the application of cold water, particularly in burns from explosions in coal mines, those injuries being attended with large quantities of coal dust and slack, forced into the patient's skin, which the cold water washes away, and gives the practitioner a better opportunity of seeing the extent of the injury.

" My reason for adding a small quantity of saturnine extract to the water, is merely to give it the appearance of a medicinal application; otherwise my patients would not have the same confidence in it; I have frequently used vinegar with advantage.

" Dr.

"Dr. Kentish appears to me to write more from theory than practice; had I been as fond of writing as he is, I could have given the public a large volume of cases; but having for a series of years been uniform in my practice, from the success attending it, I am not disposed to enter into any disputations or controversy on the subject, which a publication might probably draw me into; I am contented with the motto of my late worthy and intimate friend Dr. Kirkland, that "One *grain* of matter of fact to a practical surgeon is worth a *pound* of reasoning.''

"I trust you will excuse the rough manner in which I have made this communication to you; it may perhaps afford you some information towards defending what you have advanced on the subject of cold. About three years ago, Dr. Beddoes (who is a native of this neighbourhood) wrote to know my method of treatment in burns and scalds, which I communicated to him; in answer to my letter, he informed me, that he had fifteen years before that time, recommended cold applications to Mr. Reynolds, the proprietor of Ketley works, with whom he is intimate."

"I remain, Sir,
"Your's respectfully,
"J. Evans, M. D."

"P. S. In burns where large escars were produced, I have always recommended the bread and milk poultice in a few hours after the cold water has been used; which has been continued until a proper suppuration had taken place.

---

### CASES.

"William Welsh, in the summer of 1799, fell into a cistern of boiling water in Ketley iron works; his back, thighs, legs and arms, were severely scalded. The accident happened about seven o'clock in the evening; he was conveyed to his lodgings adjoining the works, where he was immediately wrapped in a sheet wet with cold water, (having a small quantity of extractum saturni added to it) which, in the space of two hours, afforded him considerable ease, and by twelve o'clock that night he was totally free from pain. The skin came off all the injured parts, and the patient was obliged to lie on his breast and belly for a fortnight, his wounds were sprinkled with pulverised chalk, and dressed with pledgets of tow, spread with Kirkland's nitratum; he was well at the end of the month.

"A boy

"A boy at the Horsehay iron works, near Colebrook Dale, had his leg severely scalded from his knee to his instep; *cold* water was constantly applied to the limb for the space of a week, which cured it perfectly, without any other application.

"A young man of the name of Toft, had his leg extensively scalded in Ketley works; oil of turpentine was applied to it for the space of two hours, without the least mitigation of his pain. Upon which, *cold* weak saturnine water was substituted, and in the space of a quarter of an hour he became perfectly easy. It was continued for several days until a suppuration came on; the cure was finished with powdered chalk and the neutralized cerate.

"A boy of the name of Socket, six years of age, fell into an iron boiler containing a small quantity of water, by which his arm was scalded from his shoulder to his fingers' ends. The whole arm was stript of its skin, except the back part above the elbow, which was burnt to an escar, against the side of the boiler. Linen rags wet with cold water, were applied to the parts, which afforded ease in the space of half an hour. He was treated as above, and cured in a month.

"Thomas Parish, a collier, had in August, 1800, both his hands, arms, and face severely burnt by inflammable air taking fire in a coal mine; he was treated in the same way, and found similar ease. He got well in a fortnight.

"August, 1800. Richard Price, a workman at Ketley, slipt his leg into a cistern of boiling water; he immersed his limb into *cold* water, which afforded him *immediate* ease. The same application was continued for forty-eight hours; afterwards, (the loose skin being removed) the neutralized cerate was applied, but not finding so much ease from that application, the *cold* water was again applied every day, for the space of a fortnight, which perfectly cured him.

"In October, 1800. Three boys had their faces and hands severely burnt by the explosion of hydrogen gas in a coal mine, and were treated as above, with the same success.

"October 31, 1800. A young man and boy each slipt into a cistern of boiling water in Ketley works, scalded their legs very much, and were both immediately relieved from pain by the application of cold water.

"February 21, 1801. A collier had his face, back, and shoulders burnt in a coal mine by hydrogen gas taking fire. The cold application relieved the pain in less than an hour hour. Very few blisters and little loss of skin followed the accident.

"May 21, 1801. Richard Fieldhouse had both his arms, shoulders, and upper part of his back burnt in a coal mine, by the explosion of hydrogen gas; the cold application gave him such immediate relief, that he was prevailed on to continue the use of it for a week. He was cured and able to return to his work in ten days from the time of the accident.

"June 18, 1801. John Woolley had both his arms, hands, and face, severely burnt in a coal mine; the cold application afforded perfect ease in the space of half an hour.

"June 24, 1801. Edward Jones, a young man, was carrying a pot full of boiling pitch, and spilt a small quantity of it on his hands and legs, by which he was severely scalded. After being about two hours in great pain he was relieved in a quarter of an hour by the application of cold water.

"October 30, 1801. Joseph Wilkes, had his face, shoulders, arms, and part of his back, burnt in a coal mine. The cold application gave him immediate ease, it was used for twenty-four hours; as soon as the suppuration came on, the parts were dressed with powdered chalk and Kirkland's neutralized cerate spread on tow. The face and hands had no other application than cold water, which never failed mitigating the pain, and these parts were cured in the space of a week. The back, arms, and shoulders were healed in a fortnight.

"June 14, 1803. Humphrey Thomas slipped into a cistern of boiling water in Ketley Works; he was scalded from his toes to his knees. He had no other application than cold water from the day of the accident to the 27th of the same month; by which means his legs were perfectly cured. Two small ulcers remained on the feet, which were healed by the 11th of July.

"June 22, 1805. Thomas Green, aged 24, had his face, both arms, and his back, from the upper part of the neck to the lower extremity of the spine, severely burnt in a coal mine. The cold application was made use of for 24 hours, attended with the usual success of procuring a remission of pain. When the suppuration came on, the parts were dressed as usual, and in a few days every particle of skin separated from the injured parts, leaving an exposed surface that required three yards of pledget, half a yard in breadth, to cover it. The suppuration becoming considerable, considerable, I directed the parts to be well dabbed with a strong decoction of oak bark, previous to their being sprinkled with chalk, (the latter was applied by means of a dredger commonly used for flour). In thirty days the patient was cured, the acceleration of which I attribute to the oak bark, as the discharge diminished rapidly after the use of it."

---

*Cases extracted from* Dr. Stock's *" Medical Collections on the Effects of Cold.*

" In the winter of 1797—1798, the lady of the Commandant, on Governor's Island, in the neighbourhood of New-York, inadvertently burned one of the fingers the whole length, against an iron. The surgeon of the garrison, who was present, immediately caused it to be placed in cold water, which was frequently applied for a few hours, and the burn was entirely cured.

" In the same season, some of the people of the garrison were employed in killing hogs; and a large quantity of hot water was prepared as usual, to scald them. By accident a pail full of this scalding water was thrown over the foot of a soldier; cold water was immediately dashed over it, and frequently renewed, during the space of half an hour, when he was able to proceed with his business.

" Many years ago (the reporter received the facts from the persons concerned) two brothers, apprentices to a hatter, were employed in taking hats from a boiler, and rinsing them out in a very large tub of cold water. Some dispute arising, one of them lifted the other in his arms, and seated him directly in the boiler, but being instantly struck with terror at what he had done, without loosing his hold, he again lifted him from the boiler, and seated him in the tub of cold water. The youth who had been thus hurried through these extremes of temperature, had on a pair of wide linen trowsers, and received no other injury than a narrow blister, which was formed directly under the waistband, and encircled his body.*

" The advantages derived from the use of spirits of turpentine, have been particularly insisted upon in cases of injury from explosions of gun-powder; one case has occurred within the knowledge of the author, where the liberal use of cold water was attended with a result equally satisfactory. The son of a gentleman of eminence in the

---

" * New-York Medical Repository, Vol. i. p. 538."

law department of the United States, was amusing himself with some fire-works, and in consequence of a sudden explosion, had his face and breast terribly scorched. Medical assistance was immediately obtained, and he was directed to be undrest, and laid upon the sacking of a bed. In this situation, cold water was copiously applied for a considerable time. The pain was relieved, the patient fell asleep, and awoke in the morning with no other injury than a slight superficial redness, which soon entirely disappeared. The advantages of the potatoe poultice are familiar to almost every ones experience, and they certainly arise from its long continued abstraction of the heat of the injured part."

---

It may be fairly presumed, that several of the cases here detailed, present strong instances in refutation of the hypothesis, that a burnt part requires additional, or at least, an unremitted continuance of violent excitement, to restore it to the healthful state. If such treatment were indispensably necessary, the want of it in several of the above examples, must have been conspicuous in the aggravated, nay, mortal effects of the unremedied mischief. Instead, however, of the ideal aggravation of the evil insisted on by Dr. Kentish, it appears that diminished heat at once affords ease, and expedites the cure of the injury. It may be justly supposed, that some of the violences from fire, here recorded, were sufficiently extensive to have sunk the vital power of the system to a destructive ebb, according to the theory proposed by Dr. Kentish, if unresisted by stimulating applications; yet no example is given of either general or local obstacles having arisen to the eventual welfare of the patient from the refrigerating or sedative treatment which was adopted.

From these facts it may be confidently concluded, that Dr. Kentish's theory is more visionary than practical, and that it by no means authorises the unqualified censure he has so unsparingly passed on the cooling mode of remedying injuries from fire.

( To be continued. )

